# Supplementary material for: Assessing Stress Induced by Fluid Shifts and Reduced Cerebral Clearance during Robotic-Assisted Laparoscopic Radical Prostatectomy under Trendelenburg Positioning (UroTreND Study)
Source: Methods Protoc. 2024 Apr 1;7(2):31. doi: 10.3390/mps7020031 (PMC11054176; doi:10.3390/mps7020031)
Supplement: Supplementary file 1 [file mps-07-00031-s001.zip › Suppl Figure S2_CST_V1.0_English.pdf]

LMU KLINIKUM
Klinik für Anaesthesiologie
Labor FG02 „Translationale Forschung Stress & Immunität

Current Stress Test (CST)

Code-No. (to be filled in by the investigator):

Please mark each question with a cross to indicate how you feel at the moment.
Mark each question with just one cross.

EXAMPLE:
At the moment I feel/I am

	very	quite	rather	rather	quite	very	
**fresh**	☐	☐	☐	☒	☐	☐	**dull**
**full of energy**	☐	☐	☐	☐	☒	☐	**powerless**

At the moment I feel/I am

	very	quite	rather	rather	quite	very	
**tense**	☐	☐	☐	☐	☐	☐	**composed**
**relaxed**	☐	☐	☐	☐	☐	☐	**anxious**
**concerned**	☐	☐	☐	☐	☐	☐	**carefree**
**calm**	☐	☐	☐	☐	☐	☐	**restless**
**skeptical**	☐	☐	☐	☐	☐	☐	**confident**
**comfortable**	☐	☐	☐	☐	☐	☐	**unwell**
